# Experiences of Irish Mentors and Mentees Engaged in a National Nursing and Midwifery Mentorship Programme: Mixed Methods Study With a Qualitative Focus on Mentors' Views

**DOI:** 10.1111/jan.70026

**Published:** 2025-06-20

**Authors:** Martina Giltenane, Louise Murphy, Anna Chatzi, Claire Mcnamara, Lorelli Nowell, Marie Kilduff, Aoife Lane, Margaret Williams, Owen Doody

**Affiliations:** ^1^ Health Research Institute, School of Nursing & Midwifery, Faculty of EHS, Health Sciences Building University of Limerick Limerick Ireland; ^2^ School of Nursing & Midwifery, Faculty of EHS, Health Sciences Building University of Limerick Limerick Ireland; ^3^ Faculty of Nursing University of Calgary Calgary Canada; ^4^ National Clinical Leadership Centre for Nursing and Midwifery, Health Service Executive Dublin Ireland

**Keywords:** mentees, mentors, mentorship programme, midwifery, mixed‐methods, nursing, professional development

## Abstract

**Aim:**

To gain an understanding of the experiences of mentors and mentees engaging in a national mentoring programme within nursing and midwifery in Ireland.

**Design:**

A two‐phased convergent parallel mixed methods study was undertaken.

**Methods:**

The first phase was a quantitative non‐experimental descriptive study using an online survey with mentors (*n* = 12) and mentees (*n* = 6). The second phase was a qualitative descriptive study and involved focus group discussions with mentors (*n* = 5). No mentees took part in the focus group discussions. There was a disproportionate representation of mentors versus mentees in the total sample across both phases of this study. Data were collected between December 2023 and April 2024.

**Results:**

Mentorship has a positive impact on professional growth, job satisfaction and career development for both mentors and mentees in nursing and midwifery professions. Significant challenges to effective nursing and midwifery mentorship include time constraints, irregular work patterns and a need for additional managerial and structural support. Areas identified for improvement in programme implementation include clearly defined roles, dedicated time and space for mentorship meetings and tailored support systems to address cultural diversity.

**Conclusion:**

This study highlights the significant benefits of a national formal mentorship programme; however, substantial barriers continue to underscore the need for strategic improvements. Addressing these challenges through clearer role definitions, dedicated protected mentorship time and culturally responsive support systems may enhance mentorship programme effectiveness and ensure long‐term sustainability.

**Patient or Public Contribution:**

None.

**Summary**

- Implications for the profession and/or patient care
  ○ Mentorship for nurses and midwives enhances professional development and job satisfaction, contributing to a stable nursing and midwifery workforce.
  ○ Mentorship results in increased confidence and enhanced competency which is likely to result in improved nursing and midwifery practice and better patient outcomes.
- Impact
  ○ Mentorship programmes that are effectively implemented, resourced and supported in healthcare can improve job satisfaction, skills and competence among nurses and midwives.
  ○ These results can be used by policymakers responsible for mentorship programme development and implementation to advocate for mentorship as a strategic investment, potentially influencing policies related to professional development.
  ○ Structured mentorship can reduce organisational costs by improving job satisfaction and professional growth, reducing costs associated with recruitment and training, ultimately improving the quality of patient care.
- What problem did the study address?
  ○ This study comprises an evaluation of a national nursing and midwifery mentorship programme for new graduates and junior nurses and midwives.
  ○ The experiences of nurses and midwives engaging in a national mentorship programme can guide future developments of the programme.
- What were the main findings?
  ○ Mentorship has a positive impact on professional growth, job satisfaction and career development for both mentors and mentees.
  ○ Significant challenges to effective mentorship include time constraints, irregular work patterns and the requirement for additional managerial and structural support.
  ○ Areas for improvement in programme implementation include clearly defined roles, dedicated time and space for mentorship meetings and tailored support systems to address cultural diversity and challenging communication.
- Where and on whom will the research have an impact?
  ○ This research has implications for nursing and midwifery mentors and mentees.
  ○ The results can inform mentorship programme design and policy, and policy‐makers responsible for professional development.
  ○ Junior nurses and midwives and their mentors are likely to be the main beneficiaries, together with their organisations.
  ○ The quality of patient care could improve where mentorship has improved confidence and skills in the nursing and midwifery professions.

## 1 Introduction

The Irish healthcare system faces numerous challenges as it strives to implement Sláintecare (Government of Ireland 2021); a framework designed to provide equitable, high‐quality and accessible care for all. Central to this mission is the role of nurses and midwives, who are pivotal in delivering evidence‐based, safe and patient‐centred nursing and midwifery care. However, achieving this level of care while fostering ongoing professional development requires robust support mechanisms. Among the strategies advocated to enable healthcare professionals to meet these demands is mentorship—a well‐established approach, recognised for its potential to enhance professional competence, personal development and overall job satisfaction (Health Service Executive [HSE] 2022; Vlerick et al. 2024).

## 2 Background

Mentorship in healthcare is defined as a dynamic and nurturing relationship wherein a more experienced individual supports a less experienced colleague to achieve their personal and professional goals (Nowell et al. 2017; Vlerick et al. 2024). This relationship fosters the transfer of knowledge, skills and guidance in a collaborative environment. Effective mentorship goes beyond hierarchical supervision, emphasising mutual respect, encouragement and the creation of a supportive space for growth and transition (Tiew et al. 2017; Noble 2021). For nurses and midwives, mentorship serves as a crucial bridge between academic preparation and the complex realities of clinical practice, particularly for newly graduated professionals entering an intense and often overwhelming work environment (Edwards et al. 2015; Djiovanis 2023; Moon et al. 2024).

The transition from education to practice is a well‐documented stressor for graduate nurses, characterised by challenges such as long working hours, feelings of inadequacy and adapting to complex healthcare environments (Noble 2021). These factors contribute to high levels of stress, turnover and attrition, and mentorship has emerged as a solution to these issues, offering emotional and professional support, facilitating the acquisition of clinical skills and fostering confidence and resilience (Hopkinson et al. 2023; Moon et al. 2024). Internationally, mentorship programmes have been shown to improve retention, reduce burnout and enhance both individual and organisational outcomes (Nowell et al. 2017; Moon et al. 2024). The importance of mentorship extends beyond new graduates, encompassing nurses and midwives at all stages of their careers. Structured mentoring programmes that are tailored to individual needs, foster mutual respect and emphasise that open communication and feedback are integral to their success (Fox and Champion 2022; Vlerick et al. 2024).

The benefits of mentorship in nursing and midwifery are well‐documented and have been associated with improved skill acquisition, confidence and career progression for mentees, as well as career satisfaction and personal growth for mentors (Baker et al. 2024). Mentees often report enhanced readiness for advanced roles, higher job satisfaction and better integration into clinical teams (Vlerick et al. 2024). Moreover, mentorship contributes to improved patient care outcomes by enhancing nurses' competencies in evidence‐based practice and critical care techniques (Abdullah et al. 2018; Ghosh et al. 2020).

Despite its benefits, national nursing and midwifery mentorship initiatives face several limitations that can hinder their effectiveness. Mentors and mentees often struggle to find time for mentorship due to heavy clinical workloads. Time constraints, resource limitations and balancing clinical duties with mentorship responsibilities are common barriers (Hernandez et al. 2023). The quality of mentorship can vary depending on the mentor's experience, commitment and availability. Many mentors are not formally trained, which can limit their ability to provide effective guidance (Giltenane et al. 2025). Power imbalances between mentors and mentees can inhibit open communication and learning (Wissemann et al. 2022). Ambiguity around the roles and responsibilities of mentors and mentees can lead to mismatched expectations (Wissemann et al. 2022).

Insufficient funding and administrative support can hinder the implementation and sustainability of mentorship programmes (Council and Bowers 2021).

There is often no unified framework guiding mentorship programmes, leading to variability in quality and outcomes (Giltenane et al. 2025). There is also often a lack of robust mechanisms to measure effectiveness, making it difficult to assess impact or improve practices (Wissemann et al. 2022; Giltenane et al. 2025). Giltenane et al. (2025) identified through their scoping meta‐review that many nursing and midwifery mentorship programmes are time‐limited or pilot‐based, lacking long‐term vision or integration into career development pathways. Without strong backing from leadership, mentorship programmes may not be prioritised or integrated into organisational culture (Wissemann et al. 2022). When management actively promotes mentorship by allocating time and resources, and recognises the contributions of both mentors and mentees, it signals that mentorship is a strategic priority (Giltenane et al. 2025). A supportive culture encourages open communication and mutual respect, which are key elements that enable meaningful mentor‐mentee relationships (Wissemann et al. 2022). Without such a foundation, even well‐designed mentorship programmes may not be sustainable (Wissemann et al. 2022; Giltenane et al. 2025).

Thus, the success of mentorship programmes depends on several factors, including the structure of the programme, mentor training and organisational support (Mínguez Moreno et al. 2023).

**FIGURE 1:**
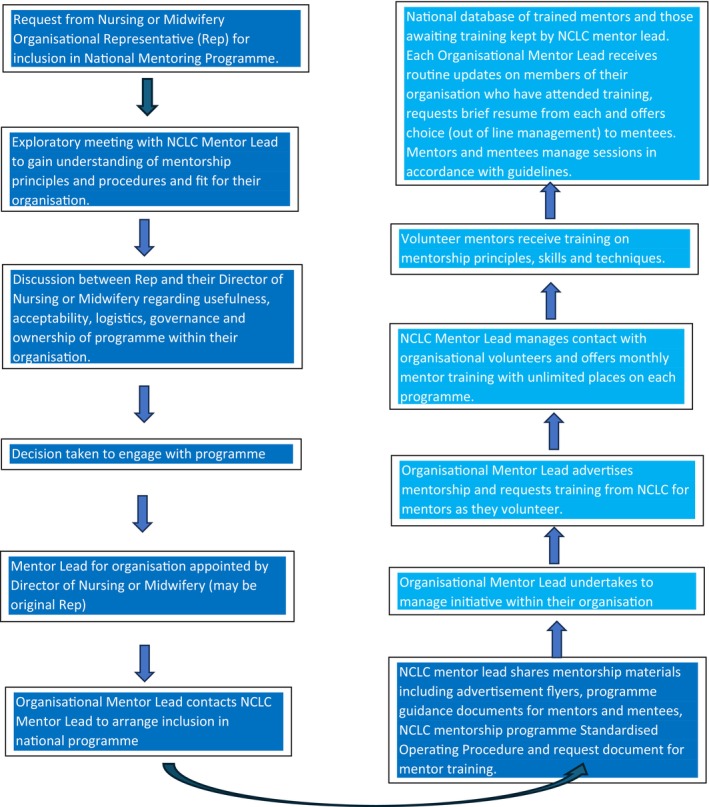
Process for implementing a national mentorship programme (Giltenane et al. 2024).

As Ireland continues to navigate the challenges of healthcare reform, mentorship stands out as a critical strategy to support the nursing and midwifery workforce. By fostering professional growth, enhancing job satisfaction and improving patient care outcomes, mentorship contributes to the resilience and sustainability of the healthcare system. In Ireland, mentorship gained momentum through initiatives such as the National Clinical Leadership Centre for Nursing and Midwifery (NCLC), which has supported mentorship programmes in healthcare since 2015. The national mentoring initiative, launched in 2021 under the auspices of the Office of Nursing and Midwifery Services Director (ONMSD), Chief Nursing Officer (CNO) and Nursing and Midwifery Board of Ireland (NMBI), and delivered by the NCLC, underscores the strategic importance of mentorship in workforce development. The structured approach outlined in Figure 1 promoted by the NCLC not only addresses the professional needs of nurses and midwives but also aims to build a culture of continuous learning and leadership within healthcare organisations. After 3 years of implementation, the programme required an evaluation to assess its effectiveness and guide future improvements (HSE 2022).

## 3 Aim

The overall aim of this mixed method study was to gain an understanding of the experiences of mentors and mentees engaging in a national mentorship programme within nursing and midwifery in Ireland.

## 4 Research Question

The research question guiding this study was: What are nurses' and midwives' experiences of engaging in a national mentorship programme that can guide future developments of the programme?

## 5 Methods

### 5.1 Design

A convergent parallel mixed methods approach was used (survey and focus group discussions) to capture mentors' and mentees' experiences of engaging in this national mentorship programme (Creswell 2021). Using a convergent parallel mixed method research design, both qualitative and quantitative research methods were combined. This reduced limitations of using quantitative or qualitative research alone. By using a convergent parallel mixed method design produced a robust description and interpretation of data, making quantitative results more understandable and increased understanding of a small‐scale qualitative study. Findings from both the qualitative and quantitative strands align to support each other, strengthening the validity and credibility of the results (Creswell 2021). Findings from both phases are presented separately and narratively merged in the discussion (Alele and Malau‐Aduli 2023). This study adhered to the Consensus‐Based Checklist for Reporting of Survey Studies guidelines (Sharma et al. 2021) (Data S1) and Standards for Reporting Qualitative Research guidelines (O'Brien et al. 2014) (Data S2). The quantitative cross‐sectional study utilised a non‐experimental descriptive design (Radhakrishnan 2013) to explore the experiences of mentors and mentees, and the qualitative study utilised a descriptive design (Sandelowski 2010) to obtain rich, in‐depth insights into the mentoring experiences of participants. Qualitative descriptive methodologies in nursing research provide a broad understanding of phenomena under investigation and is an approach commonly used as the qualitative element within mixed‐methods studies (Doyle et al. 2020). Qualitative descriptive research is aligned with pragmatism where the research approach is chosen based on the aims and context of the research study (Doyle et al. 2020).

### 5.2 Participants

Twenty‐three participants from three clinical sites located in different parts of Ireland with experience of engaging in a national mentorship programme took part in this study. A purposive sample of nursing and midwifery mentors and mentees engaged in the NCLC mentorship programme was included as potential participants. Purposive sampling was used to target participants who were best placed to discuss their views and experiences of engaging in a national mentorship programme. Eighteen participants (6 mentees, 12 mentors) out of a potential 63 (28.57% response rate) responded to the survey and two focus groups were conducted with five mentors online via Microsoft Teams.

### 5.3 Recruitment

#### 5.3.1 Survey

The research team contacted designated mentor leads at the five organisations for which ethical approval was obtained. Three organisations participated. The mentor leads distributed the surveys to mentors and mentees within their organisations. All relevant information was provided, including an information package and the researcher's contact details. Informed consent was obtained before proceeding with the survey. All potential participants received an initial invitation and two‐weekly reminders to complete the survey.

#### 5.3.2 Measures and Procedures

Based on a review of the literature the mentor evaluation tool (MET) (Yukawa et al. 2020) questionnaire was chosen based on its relevant item content and high internal reliability scores. The questionnaire was developed further with input from the research team and members of the NCLC research oversight group over three rounds, to reflect the characteristics of nursing and midwifery mentorship within the Irish setting. Following this, two separate questionnaires were developed to reflect both mentors' and mentees' experiences. The questionnaire was then piloted with seven participants to identify readability, bias, clarity, layout and flow issues and minor amendments were made. The questionnaire for mentors and mentees comprised 15 and 20 items respectively. Both surveys had two open ended questions for each group and all participants had the opportunity to add comments following each closed question. Data were collected using an online survey tool between December 2023 and January 2024, and responses were coded and entered into the statistical package for the social sciences (SPSS, V28).

#### 5.3.3 Focus Groups

The purpose of the focus group discussions was to further explore the experiences of the mentors and mentees engaging in the NCLC mentoring programme. Mentors and mentees who participated in the survey had the opportunity to opt into focus group discussions. However, no respondents opted to participate through this option. After consultation with the NCLC oversight team, the mentorship leads within the participating areas were contacted by the research team and information in relation to the focus group discussions were distributed. A QR code and a link to take part in the focus groups were provided. Five mentors participated in focus group discussions, however no mentees agreed to participate. Two focus groups were conducted with mentors via Microsoft Teams in April 2024, guided by a semi‐structured interview schedule (Data S3) informed by a review of the literature and findings from the survey results. Three participants took part in the first focus group and two in the second. A focus group moderator facilitated each of the focus groups. A co‐moderator took notes, observed group dynamics and asked prompt questions as required. Written informed consent was obtained from all participants prior to focus group discussions. Researcher reflexivity is central when undertaking qualitative empirical research (Flemming and Noyes 2021). When reflexivity is not considered, it reduces empirical research quality (Flemming and Noyes 2021). All researchers have extensive experience in undertaking qualitative research and had no relationship with participants before undertaking the research. The researchers were aware of the insertion of their values and subjective ways of knowing and being in analysing and writing in the research (Ide and Beddoe 2023). The researchers were reflexive about their assumptions and biases and controlled these holding presuppositions in abeyance throughout the research process and thus situating themselves in a neutral stance (Ide and Beddoe 2023).

### 5.4 Ethical Considerations

Ethical principles outlined by the nursing and midwifery board of Ireland (NMBI 2015) were adhered to throughout the study to ensure participant safety, confidentiality and informed consent. Ethical approval was granted by the Education and Health Sciences Research Ethics Committee at the University of Limerick (Reference: 2022‐11‐31‐EHS) and two joint Clinical Research Ethics Committees covering multiple clinical sites (ECM4 & 3826).

### 5.5 Data Analysis

Descriptive statistics were used to summarise survey responses using frequencies to detail the number and percentage of responses to each question. The response rate was insufficient to support inferential statistical analysis or statistical comparisons and reach conclusive results.

**FIGURE 2:**
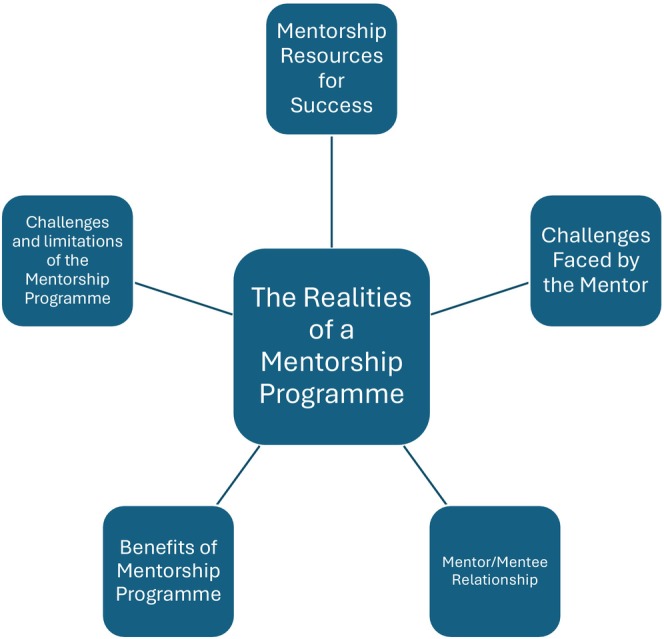
Subordinate theme and sub themes.

The focus group discussions were audio‐recorded and transcribed verbatim. Focus group findings are supported by direct quotations to allow the reader to judge the dependability. Data were de‐identified using pseudonyms. One focus group transcript (50% of transcripts) was reviewed and verified by a second coder using a coding framework based on theoretical perspectives guided by the research objectives (Attride‐Stirling 2001; Burla et al. 2008; Ezzy 2013). There was over 60% agreement between the data coders indicating a reasonable degree of reliability as per Julien et al. (2011). Thematic analysis was used and produced thematic networks; ‘web‐like illustrations that summarise the main themes constituting a piece of text’ (Attride‐Stirling 2001, 386). As outlined by Attride‐Stirling (2001) six main steps to analysis were conducted; coding the material, identifying themes, constructing the networks, describing and exploring thematic networks, summarising thematic networks and interpreting patterns. Table 1 details each step taken. Examples of moving from codes to basic themes, moving from basic to sub themes and moving from sub themes to the superordinate theme are presented in Table 2. This paper presents the emergent superordinate theme ‘The Realities of a Mentorship Programme’. Figure 2 illustrates the five sub themes. Data integration was conducted, findings from the survey and focus groups were collectively examined to derive a comprehensive understanding of the mentoring programme.

**TABLE 1:** Attride‐Stirling's (2001) six step thematic analysis.

Steps 1–6	Details for this study
Step 1: coding the material	This involved reducing the data to manageable and meaningful text sections. The coding framework was based on matters ascending from the data (Attride‐Stirling 2001). Once all the text had been coded, themes were abstracted from the coded text sections. An example of the transition of codes to basic themes is presented in Table 2.
Step 2: identifying themes	After identifying the codes, the next step was the identification of themes by reading the text sections within the context of the assigned codes. This process enabled the researcher to reframe the reading of the text, thus identifying patterns and structures from examining the coded text sections (Attride‐Stirling 2001). The themes that emerged were specific enough to connect to one idea but broad enough to show similarities in various different text sections.
Step 3: constructing the network	Constructing the network required grouping the themes into meaningful divisions, identifying the basic themes, reorganising basic themes into sub themes and deducing these themes into the superordinate theme (Attride‐Stirling 2001). The network was verified and refined according to Attride‐Stirling's (2001) guidelines by going through the text sections to ensure that superordinate theme, sub themes and basic themes were supported by the data.
Step 4: describe and explore the thematic network	Once the network was constructed the researchers went back to the original text and interpreted the text with the aid of the network. The network was described using quotations from the text to support the description. This step organised the data and the interpretation, and elaborates the analysis for the readers.
Step 5: summarise the thematic network	This step summarised the main theme characterising network (Attride‐Stirling 2001). This step served to make explicit the patterns that emerged in the exploration.
Step 6: interpret patterns	We returned to the original research objectives and the theoretical interests underpinning them in this final step and addressed these with arguments grounded on the patterns that emerged in the exploration of the texts (Attride‐Stirling 2001). Thus the focus group topic guides were used with a view to focusing on the research objectives as closely as possible.

**TABLE 2:** Example of moving from codes to basic themes, to sub themes to the superordinate theme.

Codes	Basic themes	Sub theme	Superordinate theme
Mediation Unpacking issues with Mentees Value of experience Encouragement Identifying client blocks Experienced mentor with on the ground experience Emotional Intelligence of Mentor/ability to recognise Mentee boundaries Advice and sharing of expertise Sense of satisfaction in mentor role Leadership abilities Establishing trust Finding a level of satisfaction in goals achieved Gaining resilience through achieving goals Growth mindset Holistic mentoring of mentee Identifying mentoring styles to minimise risk of personality clash Choosing mentor based on mutual interested Individual needs/expectations of mentee	Role of mentor Important mentor qualities Relationship building between mentor and mentee	Mentoring/mentee relationship	The realities of a mentorship programme

### 5.6 Research Rigour

To ensure the validity and rigour of this study, the researchers utilised the mentor evaluation tool (MET) a recognised clinical supervision tool with good reliability and wide usage (Yukawa et al. 2020).

Focus group discussions were chosen based on the aims and context of the research study (Doyle et al. 2020). Focus group discussions were recorded and transcribed verbatim; data collection methods and analysis procedures were described, direct quotations are used to support the findings, and the authors' biases were minimised throughout the research process.

Reporting rigour was demonstrated using the Consensus‐Based Checklist for Reporting of Survey Studies guidelines (Sharma et al. 2021) (Data S1) and Standards for Reporting Qualitative Research guidelines (O'Brien et al. 2014) (Data S2).

## 6 Results

### 6.1 Quantitative Data

Out of 63 active mentors and mentees identified, the team received 18 fully completed questionnaires (total response rate 28.6%). The response rate for mentees (*N* = 6) was 20.7% and for mentors (*N* = 12) was 35.3%.

### 6.2 Participant Characteristics

Mentee participants included four females, one male and one who preferred not to say. Four mentees were currently working in acute adult nursing settings and two were working as public health nurses. Mentees' ages ranged from 25 to 38 years old. Mentees participated in the mentorship programme as they were asked by managers to participate (*n* = 2), reacted to an advertisement (*n* = 1), made the decision during a preparatory/induction week (*n* = 1) and because they were considering becoming a mentor themselves (*n* = 1).

All mentor participants identified as female (*n* = 12), with between 1 and 25‐years' experience in their current role within acute adult nursing setting (*N* = 11) and public health nursing (*N* = 1). Mentors participated in the mentorship programme as they were asked to join by management or thought it was part of their job requirement (*n* = 3) or volunteered to participate as a mentor (*n* = 9). The nine mentors who volunteered became familiar with the programme through discussion with colleagues and email communication.

### 6.3 Mentees' Responses

**FIGURE 3:**
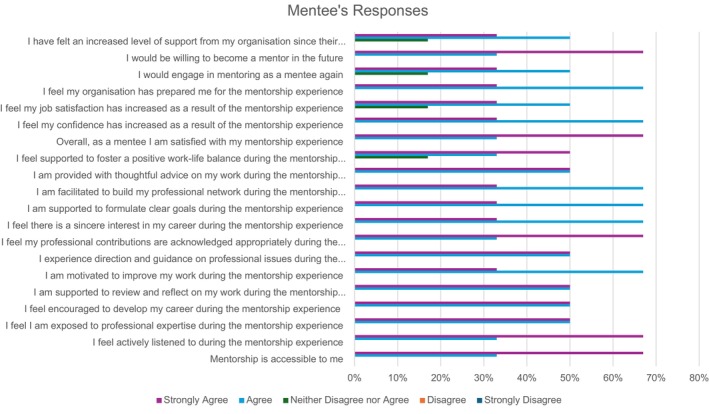
Mentees' responses to survey questions.

Overall, mentees were very satisfied with the mentorship programme, all agreed or strongly agreed that they had an increased level of support from their organisation since their commitment to the mentorship programme (Figure 3). Mentees agreed or strongly agreed that the programme was accessible, they expressed that they were listened to by their mentors and were encouraged with their career development. Mentees agreed or strongly agreed that mentors promoted exposure to professional expertise, supported their reflections and provided review toward improvement. Mentees agreed or strongly agreed that mentors gave them positive direction, thoughtful advice and guidance, showed sincere interest in them and appreciated their contribution. Mentees agreed or strongly agreed that mentors supported them in creating clear goals and networks. Mentees also agreed or strongly agreed that they would engage with the programme as mentors in the future.

Three mentees strongly agreed and two agreed that they felt supported to foster a positive work‐life balance during the mentorship experience. Three mentees agreed and two strongly agreed that their job satisfaction had improved because of the mentorship experience, and that they would engage in a mentorship programme again.

### 6.4 Mentors' Responses

**FIGURE 4:**
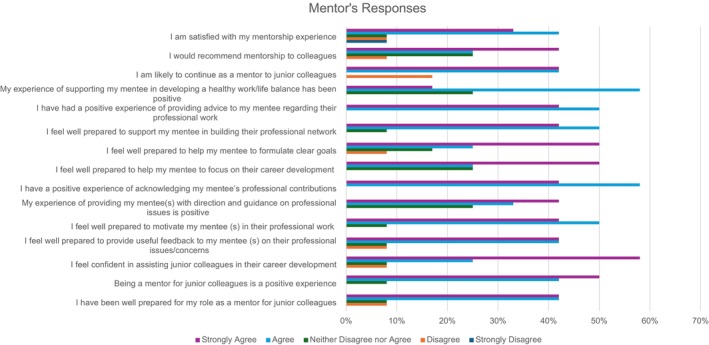
Mentor's responses to survey questions.

**FIGURE 5:**
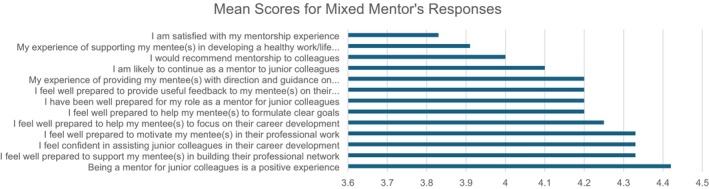
Mean scores for mixed mentor's survey responses (strongly disagree = 1 to strongly agree = 5).

Overall, mentors responded very positively to the mentorship programme questionnaire (Figure 4), however there were some mixed responses which can be seen in Figure 5. Half of the mentors agreed, and the other half strongly agreed that they appreciated mentees' professional contribution and had a positive experience in advising mentees. Most mentors (*N* = 11) either agreed or strongly agreed that being a mentor for junior colleagues is a positive experience, that they were well prepared to motivate mentee(s) in their professional work and prepared to build their professional network.

Accepting three mentors, who neither agreed nor disagreed, others (*n* = 9) either agreed or strongly agreed that their experience of providing mentee(s) with direction and guidance on professional issues was positive, that they were well prepared to help their mentee(s) focus on their career development, and that their experience of supporting their mentee(s) in developing a healthy work/life balance had been positive.

Most mentors either agreed (*n* = 5) or strongly agreed (*n* = 6) that they were well prepared to mentor junior colleagues, were confident in assisting junior colleagues in their career development and were prepared to provide useful feedback to their mentee(s) on their professional issues/concerns.

Most mentors agreed (*n* = 3) or strongly agreed (*n* = 6) that they were prepared to help their mentee(s) to formulate clear goals, and most mentors agreed (*n* = 3) or strongly agreed (*n* = 5) that they would recommend mentorship to colleagues. Likewise, most mentors agreed (*n* = 5) or strongly agreed (*n* = 5) that they are likely to continue as a mentor to junior colleagues. Most mentors (*n* = 5) agreed or strongly agreed (*n* = 4) that they are satisfied with their mentorship experience; notwithstanding its positivity, this was the least favourable response of all the survey questions. The mentors' mixed responses to survey questions are presented in Figure 5.

### 6.5 Qualitative Data Including Open Ended Survey Responses

#### 6.5.1 Demographic Details

Five participants participated in the focus group discussions, four female and one male ranging in age between 29 and 45 years old. Participants were qualified as registered general nurses between 6 and 24 years. All participants were working as general nurses in their current acute setting between one and 7 years as Clinical Nurse Managers two (CNM2) or Assistant Directors of Nursing. Two of the participants previously worked outside of Ireland. Three participants were of Irish nationality; one was from India, and one was from the Philippines. All had a minimum educational qualification of postgraduate diploma.

All participants had completed the NCLC mentorship training and were also involved in unofficial mentoring as part of their clinical role for between four and 24 months. Participants completed the NCLC mentorship programme when access to training or opportunity to train was available within their organisation. Open ended survey responses from mentors in Phase two of this study are included in the qualitative findings presented.

### 6.6 Qualitative Findings

Five sub‐themes emerged under this superordinate theme: ‘The realities of a mentorship programme’. These included ‘Mentorship Resources for Success’, ‘Challenges faced by the Mentor’, ‘Mentor/Mentee Relationship’, ‘Benefits of Mentorship Programme’ and ‘Challenges and Limitations of the Mentorship Programme’.

This superordinate theme highlights several key factors highlighting the realities of a mentorship programme in a clinical setting. Participants emphasised the need for dedicated, confidential spaces—such as a ‘mentor hub’—to support effective mentoring, as current arrangements in public or semi‐private areas were seen as inadequate. Time constraints due to clinical demands were a recurring challenge, with protected time often promised but rarely feasible in practice. Mentors noted that their motivation and effectiveness were enhanced by mentee feedback and visible progress. Challenges included mediating interpersonal conflicts, addressing cultural and communication barriers and managing personal issues affecting mentees' performance. Despite concerns about qualifications, mentors felt empowered when they understood mentees' needs and could draw on their experience. Strong mentor‐mentee relationships were linked to communication, compassion and critical thinking, though personality clashes sometimes hindered progress. Ultimately, the programme was seen as mutually beneficial. Mentors gained fulfilment and professional growth, while mentees received guidance, support and career development opportunities. Participants identified that irregular work schedules, clinical demands and personal commitments posed significant challenges to maintaining consistent engagement in the mentorship programme for both mentors and mentees. Additionally, mismatched expectations about the mentor's role and limited managerial support further hindered the effectiveness and sustainability of the programme. Each of the sub themes that encapsulate the fundamentals of this superordinate theme: ‘The realities of a mentorship programme’ are presented.

#### 6.6.1 Mentorship Resources for Success

The success of the mentorship programme was closely tied to the availability of appropriate resources, particularly dedicated space and protected time. Participants emphasised that confidentiality and consistency were compromised when sessions were held in informal or busy settings, highlighting the need for a designated ‘*mentor hub*’. Despite formal recognition of protected time for mentoring, clinical demands often made this impractical. Nonetheless, mentors found motivation in positive feedback from mentees, which reinforced the value of their role:

If you could have a mentoring hub… you wouldn't have to worry about going to the coffee shop hoping it's not too busy. (FG1, P1)

While in theory time is protected for mentorship, in reality, time is always an issue. (Survey, Mentor 13)

It's great… when they actually take your advice on board and they really come back and praise you. (FG2, P1)

#### 6.6.2 Challenges Faced by the Mentor

Mentors in the programme faced a range of challenges that extended beyond traditional guidance, often requiring them to mediate interpersonal conflicts, navigate cultural differences and support mentees through personal and professional barriers. These responsibilities demanded strong leadership and adaptability, as mentors worked to set goals and foster growth. Cultural diversity added complexity to relationship‐building, with communication styles and beliefs sometimes hindering connection. Despite initial doubts about their qualifications, mentors found that understanding mentees' needs and drawing on their own experience helped them grow in confidence and effectiveness:

Getting the two people together to find a bit of a mediation and set out goals… from a learning point of view. (FG2, P2)

Culture communication is one of the biggest… differences that we need to kind of explore. (FG2, P1)

I was glad of the opportunity to develop skills and confidence in being more effective. (Survey, Mentor 12)

#### 6.6.3 Mentor/Mentee Relationship

The mentor/mentee relationship was seen as central to the success of the mentorship programme, with effective communication, compassion and critical thinking identified as essential mentor qualities. Mentors often supported mentees through personal challenges that impacted their clinical performance, highlighting the need for emotional intelligence and adaptability. Experience and expertise were also seen as key to building trust and setting realistic goals. However, personality clashes occasionally hindered progress, reinforcing the importance of flexibility and the option for mentees to change mentors:

It is dealing with all those external problems… on top of providing clinical support and mentorship… That is probably been my biggest challenge yet. (FG2, P2)

You have ten plus years of experience… a person who's probably new to that area would actually gain a lot. (FG1, P2)

They didn't even complete the process because they said there was a bit of clash of personality. (FG1, P1)

#### 6.6.4 Benefits of Mentorship Programme

The mentorship programme was seen as highly beneficial for all stakeholders, offering personal and professional growth for mentees, job satisfaction for mentors and broader organisational advantages such as staff retention and career development. Mentors found the role deeply rewarding, while mentees benefited from tailored guidance on career progression. Additionally, mentors actively facilitated networking by connecting mentees with professionals aligned to their goals:

It's a very fulfilling kind of a role. (FG1, P1)

(Mentee) reached out to me about career progression and ways that they could enhance their development here in the organisation. (FG1, P1)

If somebody would like to go an academic path… I would guide them to a person… who would be a better fit. (FG1, P2)

#### 6.6.5 Challenges and Limitations of the Mentorship Programme

The mentorship programme faced several challenges and limitations, primarily due to the demanding and unpredictable nature of clinical work, which affected both mentors' and mentees' ability to consistently engage. Irregular schedules, shift work and competing responsibilities often led to missed or rescheduled meetings. Additionally, mentors encountered unrealistic expectations from mentees, such as assuming mentors could secure university placements or funding. Participants also highlighted the need for stronger managerial support to protect mentorship time and ensure the programme's sustainability:

People might have arranged a meeting with me and then had to cancel because something popped up in the clinical area. (FG1, P1)

That just wasn't within my realm or scope to do. (FG1, P1)

There needs to be stronger buy‐in from management. (Survey, Mentor 13)

## 7 Discussion

This study provides critical insights into the experiences of nurses and midwives participating in a national mentorship programme in Ireland. The findings emphasise the integral role of mentorship in fostering professional development, job satisfaction and improved patient care outcomes, consistent with international literature on mentorship in healthcare (Burgess et al. 2018; Hookmani et al. 2021). The data reveal that mentorship strengthens professional relationships, enhances career satisfaction and supports mentees' career progression. Both mentors and mentees highlighted the significance of mentorship in building confidence, developing clinical skills and enhancing resilience, corroborating studies indicating that mentorship facilitates advanced role readiness and career advancement (Hagrass et al. 2023; Mudderman et al. 2020).

Mentors frequently reported professional fulfilment and an enhanced sense of purpose, mirroring research that links mentorship to personal growth and satisfaction (Dopson et al. 2017). Similarly, mentees reported increased job satisfaction, enhanced support and a desire to serve as future mentors, indicating the programme's potential for enduring impact within the healthcare system. While the NCLC mentorship programme was a voluntary programme fully supported by Directors of Nursing and Midwifery to implement within their organisations, challenges such as time constraints, competing clinical priorities and the need for additional managerial support were noted as significant barriers to effective mentorship. These challenges align with existing literature, which underscores the necessity for organisational investment in mentorship resources and support (Manzi et al. 2017; Rohatinsky et al. 2018). Cultural differences, challenging communication and personality mismatches also emerged as barriers. This underscores the usefulness of a chemistry meeting and the potential benefits of additional initiatives to support inclusivity and cultural competence within organisations (Luukkonen et al. 2023).

Participants advocated for protected time for mentorship activities and dedicated mentorship spaces. Addressing these barriers by creating mentor hubs and providing protected time, can foster an environment conducive to successful mentorship. These recommendations are consistent with evidence promoting structured and well‐supported mentorship programmes to maximise their effectiveness (Chan et al. 2020). The sustainability of such programmes hinges on robust mentor training and management support (Chan et al. 2020).

Our study also identified the importance of role‐clarity and mentors setting boundaries with mentees in relation to their role. Mínguez Moreno et al. (2023) recommend that mentor preparation should include training in effective role management and communication of these roles. This will ensure mentors maintain professionalism and clarity in their roles. Equipping mentors with professional development opportunities and clear role definitions enhances their confidence and efficacy (Gazaway et al. 2019; Al Habeeb et al. 2024) which was clear from participant quotations in our study.

Engaging in a national mentorship programme for nurses and midwives in Ireland provides valuable insights into the multidimensional impact of mentorship on professional development, knowledge sharing and workplace culture. In a study by Hoover et al. (2020), mentors frequently reported growth and fulfilment as they reflected on their practices while nurturing the next generation of healthcare professionals which is similar to findings of our study. For mentees, according to Gopee (2015), mentorship programmes bridge the gap between theoretical knowledge and clinical practice, offering critical support during transitions from academic to professional environments.

Despite these benefits, challenges persist. Mentors often cited time constraints and workload pressures as obstacles to effective mentoring, which can result in variable experiences for mentees (Cross et al. 2019). Our study identified that mentees sometimes hesitated to engage fully. Others have reported that this may be due to concerns about being perceived as burdensome or dependent (Zhang et al. 2023). National mentorship frameworks in Ireland aim to address these issues by encouraging structured training, fostering open communication and cultivating supportive relationships.

The dual role of mentorship in fostering professional competence and creating a community of practice, highlights the importance of institutional support and resource allocation for mentorship initiatives (National Leadership and Innovation Centre for Nursing and Midwifery 2018). These findings contribute to a broader understanding of mentorship as a transformative process that not only develops individual skills but also strengthens the profession's capacity to adapt to evolving healthcare demands.

The strategic significance of mentorship in workforce development is particularly relevant as the Irish healthcare system implements Sláintecare reforms. By addressing identified barriers and building on the programme's strengths, mentorship can serve as a cornerstone strategy for improving staff retention, professional growth and patient care quality. Policy efforts should prioritise embedding mentorship into organisational culture and ensuring adequate resources for its long‐term success.

Although our study is situated within the Irish healthcare system, the findings resonate with global literature on mentorship. For example, mentorship programmes in Canada (Nowell et al. 2017), South Korea (Moon et al. 2024) and Sub‐Saharan Africa (Manzi et al. 2017) have highlighted similar challenges regarding organisational support, protected time and programme sustainability. Notably, the importance of cultural competence and structured mentor training is echoed in cross‐cultural studies such as Luukkonen et al. (2023), which found that mentor preparedness significantly affects mentorship outcomes in diverse clinical settings. These comparisons highlight how the Irish mentorship experience aligns with and diverges from global efforts and underscore the universal need for robust support structures and culturally responsive approaches.

While many of the identified benefits and barriers align with established mentorship literature, a key theoretical contribution of this study is its emphasis on the contextual dependency of mentorship success. Specifically, this study highlights that even well‐designed mentorship programmes are unlikely to succeed without alignment with clinical workflows, managerial buy‐in and protected time a—reflection of the pragmatic theory of implementation (Doyle et al. 2020). Moreover, the data suggest that mentorship may function not just as a developmental relationship but as a mechanism for cultivating a resilient professional identity within complex, resource‐limited systems. This extends beyond conventional role modelling frameworks by positioning mentorship as a strategy for organisational adaptation and workforce retention.

It must be noted that while our findings highlight the potential benefits of mentorship programmes in enhancing job satisfaction and professional development, these insights must be interpreted with caution given the small sample size and limited mentee representation. The qualitative strand included only five mentors, and no mentees participated in focus groups, which restricts the breadth of experiences captured. It must be explicitly noted that the absence of mentee voices in the qualitative phase of this mixed method study is a major limitation affecting data triangulation and interpretation. As such, the conclusions drawn from the qualitative data are tentative and may overrepresent mentor perspectives. Rather than generalising, our intention is to offer preliminary insights that can inform future, larger‐scale evaluations.

### 7.1 Limitations

While this study provides valuable insights into the experiences of mentors and mentees engaging in the National Mentorship Programme for nursing and midwifery in Ireland, several limitations should be acknowledged. Firstly, the response rate of the quantitative phase of this study was 28.6%, with only 18 participants completing the survey. Access to participants within the timeframe of the funded research project was difficult as currently there is no National Research Ethics Committee that incorporates all health service organisations. Most organisations have individual Research Ethics Committees and did not accept ethical approval from the research team's institute. Therefore, a pragmatic decision was made to gain research ethical approval from organisations that had the most participation in the mentorship programme or incorporated multiple organisations within their research approval. The low response rate significantly limits the reliability and generalisability of quantitative findings and the statistical power of the quantitative data. Secondly, the majority of survey respondents were mentors (*n* = 12), with a smaller representation from mentees (*n* = 6). A larger representation from mentees could provide different results. Thirdly, while the Mentor Evaluation Tool (MET) was adapted for the study, its psychometric properties were not tested within the Irish context due to the low sample size, critically limiting the ability to confirm its reliability and validity in this setting. Fourthly, the focus groups were conducted exclusively with only five mentors meaning a thin data set limiting the findings to one superordinate theme. This is a major limitation which reduces the depth of the findings and may have introduced a bias toward mentor‐centric perspectives. Mentee‐specific challenges and insights remain underexplored. Fifthly, the study relied on voluntary participation, which may have introduced self‐selection bias, as participants who were more positively inclined toward the mentorship programme might have been more likely to participate. Sixthly, the study's cross‐sectional design provides a snapshot of participants' experiences at a single point in time, limiting the ability to assess longitudinal outcomes or changes over time. Additionally, participants were not given the opportunity to review or validate the interpretations of their responses, which may affect the credibility of the findings. Finally, the study focused on a specific national mentorship programme in Ireland, which may limit the applicability of the findings to other contexts or healthcare systems.

## 8 Conclusion

This study offers valuable insights into the experiences of nurses and midwives engaging in a national mentorship programme within the Irish healthcare system. The findings highlight the positive impact of mentorship on professional growth, job satisfaction and career development for both mentors and mentees. Participants reported numerous benefits, including improved support structures, enhanced career progression opportunities and strengthened professional relationships. However, significant challenges were also identified. Time constraints, irregular work patterns and the requirement for additional managerial and structural support emerged as key barriers to effective mentorship. The lack of dedicated spaces for mentoring sessions and the competing demands of clinical duties were noted as inhibiting factors that need to be addressed for the programme's sustainability and success. Moreover, the study revealed areas for improvement in programme implementation, such as the need for clearly defined roles and tailored support systems to address cultural diversity. While the study highlights the importance of mentorship in supporting Ireland's nursing and midwifery work amid ongoing reforms, it also emphasises the need for strategic investment in resources, organisational support and programme evaluation. Future developments should focus on addressing the identified limitations, enhancing programme structure and fostering a culture of continuous learning and leadership. Ultimately, this research reaffirms mentorship as a transformative tool for professional development in nursing and midwifery, while advocating for systemic changes to optimise its effectiveness and long‐term impact within the Irish healthcare system.

## Author Contributions

All authors have agreed on the final version and meet at least one of the following criteria (recommended by the ICMJE*): (1) substantial contributions to conception and design, acquisition of data or analysis and interpretation of data; (2) drafting the article or revising it critically for important intellectual content.

## Conflicts of Interest

The authors declare no conflicts of interest.

## Peer Review

The peer review history for this article is available at https://www.webofscience.com/api/gateway/wos/peer‐review/10.1111/jan.70026.

## Supporting information


Data S1.

Data S2.

Data S3.

## Data Availability

The data that support the findings of this study are available from the corresponding author upon reasonable request.
