# Supplementary material for: Experiences of Irish Mentors and Mentees Engaged in a National Nursing and Midwifery Mentorship Programme: Mixed Methods Study With a Qualitative Focus on Mentors' Views
Source: J Adv Nurs. 2025 Jun 20;81(11):7978–91. doi: 10.1111/jan.70026 (PMC12535331; doi:10.1111/jan.70026)
Supplement: Supplementary file 3 — Data S3. [file JAN-81-7978-s001.docx]

**Supplementary File 3 Focus Group Interview Schedule**

**Focus Group Interview Schedule for Mentors engaging in a National Mentoring Programme.**

**Briefing**

Introduction of the Researcher/Facilitators

Study Explained

Participant informed about anonymity, confidentiality and recording the interview

Questions invited from participant

Date: Venue: Online Time:

1. Can you tell me about your experience of working as a Mentor?
2. Can you tell me about the challenges you have encountered working as a mentor?
3. Can you tell me about the benefits of being a mentor
4. Can you tell me about the benefits of mentorship for the mentees?
5. Can you tell me about the disadvantages of working as a mentor?
6. Is there anything else you would like to add?

**Debriefing**

Do you have any questions you would like to ask?

Thank you for your time and participation in this study.

**Probes**

Can you think of any examples or stories that explain that?

You mentioned _________ what do you mean by that/can you elaborate/explain further?

Have you had experience of that?
